# NAD+ Attenuates Bilirubin-Induced Hyperexcitation in the Ventral Cochlear Nucleus by Inhibiting Excitatory Neurotransmission and Neuronal Excitability

**DOI:** 10.3389/fncel.2017.00021

**Published:** 2017-02-03

**Authors:** Min Liang, Xin-Lu Yin, Lu-Yang Wang, Wei-Hai Yin, Ning-Ying Song, Hai-Bo Shi, Chun-Yan Li, Shan-Kai Yin

**Affiliations:** ^1^Department of Otorhinolaryngology, The Sixth People’s Hospital, Shanghai Jiao Tong UniversityShanghai, China; ^2^Programs in Neurosciences and Mental Health, SickKids Research Institute and Department of Physiology, University of TorontoToronto, ON, Canada; ^3^Med-X Research Institute and School of Biomedical Engineering, Shanghai Jiao Tong University ShanghaiShanghai, China; ^4^Department of Otorhinolaryngology, West China Hospital, Sichuan UniversityChengdu, China

**Keywords:** nicotinamide adenine dinucleotide (NAD+), ventral cochlear nucleus, patch-clamp recording, miniature excitatory postsynaptic currents (mEPSCs), evoked EPSCs (eEPSCs), spontaneous spike, bilirubin encephalopathy, hyperexcitation

## Abstract

Nicotinamide adenine dinucleotide (NAD+) is an important molecule with extensive biological functions in various cellular processes, including protection against cell injuries. However, little is known regarding the roles of NAD+ in neuronal excitation and excitotoxicity associated with many neurodegenerative disorders and diseases. Using patch-clamp recordings, we studied its potential effects on principal neurons in the ventral cochlear nucleus (VCN), which is particularly vulnerable to bilirubin excitotoxicity. We found that NAD+ effectively decreased the size of evoked excitatory postsynaptic currents (eEPSCs), increased paired-pulse ratio (PPR) and reversed the effect of bilirubin on eEPSCs, implicating its inhibitory effects on the presynaptic release probability (Pr). Moreover, NAD+ not only decreased the basal frequency of miniature EPSCs (mEPSCs), but also reversed bilirubin-induced increases in the frequency of mEPSCs without affecting their amplitude under either condition. Furthermore, we found that NAD+ decreased the frequency of spontaneous firing of VCN neurons as well as bilirubin-induced increases in firing frequency. Whole-cell current-clamp recordings showed that NAD+ could directly decrease the intrinsic excitability of VCN neurons in the presence of synaptic blockers, suggesting NAD+ exerts its actions in both presynaptic and postsynaptic loci. Consistent with these observations, we found that the latency of the first postsynaptic spike triggered by high-frequency train stimulation of presynaptic afferents (i.e., the auditory nerve) was prolonged by NAD+. These results collectively indicate that NAD+ suppresses presynaptic transmitter release and postsynaptic excitability, jointly weakening excitatory neurotransmission. Our findings provide a basis for the exploration of NAD+ for the prevention and treatment of bilirubin encephalopathy and excitotoxicity associated with other neurological disorders.

## Introduction

Emerging evidence indicates that nicotinamide adenine dinucleotide (NAD+) plays essential roles in energy metabolism, mitochondrial functions, aging, calcium homeostasis, immune functions and gene expression (Ying, 2006, 2008; Xia et al., 2009). A series of studies have shown that NAD+ treatment can significantly decrease cell death induced by oxidative stress (Alano et al., 2004), oxygen-glucose deprivation (Wang et al., 2008), genotoxic agents and zinc (Cai et al., 2006). Despite the wide range of biological functions of NAD+ in cellular processes, little attention has been paid to the action of NAD+ on neuronal excitation and its protective effect on excitotoxicity, which is closely related to neuronal injury in many neurodegenerative diseases. A previous study showed that poly(ADP-ribose) polymerase-1 (PARP-1), a major NAD+-consuming enzyme, is involved in glutamate NMDA receptor-dependent neuronal death (Cosi et al., 2004). Moreover, NAD+ was implicated in inhibiting apoptotic neuronal death after glutamate insult (Wang et al., 2014), implicating the potential of NAD+ in protecting neurons from excitotoxicity under pathological conditions.

Excitotoxicity was proposed as one of the major mechanisms underlying bilirubin (Bil) encephalopathy (Watchko, 2006; Watchko and Tiribelli, 2013), a devastating neurological disorder that results from preferential bilirubin toxicity in neurons, with a regional topography of injuries to specific nuclei in the central nervous system (CNS; Ostrow et al., 2003). Our previous studies showed that bilirubin could induce neuronal hyperexcitation via the potentiation of presynaptic glutamate release in the ventral cochlear nucleus (VCN) and lateral superior olive nucleus (LSO), which are most vulnerable to bilirubin (Li et al., 2011, 2012). It is known that glutamate excitotoxicity is linked to bilirubin encephalopathy, which can be attributed to the deposition of unconjugated bilirubin in selective brain areas during neonatal hyperbilirubinemia. However, there are no effective pharmacological treatments to alleviate the toxic effects associated with hyperbilirubinemia.

To explore the potential utility of NAD+ for neuroprotection, we made patch-clamp recordings from VCN neurons in acute auditory brainstem slices from early postnatal rats (<2 weeks old), and investigated the effects of NAD+ on synaptic transmission and neuronal excitability with or without addition of bilirubin to simulate neonatal hyperbilirubinemia. Our results demonstrated that NAD+ inhibits presynaptic glutamate release and downregulates the intrinsic excitability of postsynaptic neurons, ultimately attenuating neuronal excitation. More importantly, NAD+ reversed and prevented bilirubin-induced hyperexcitation in VCN neurons.

## Materials and Methods

### Ethics Statement

Experiments were conducted in conformity with the guiding principles for the care and use of animals, and experimental protocols were approved by the Ethics Review Committee for Animal Experimentation of Shanghai Jiao Tong University. All protocols were developed in accordance with the National Institutes of Health guide for the care and use of laboratory animals. Throughout the experiment, all efforts were made to alleviate animal suffering.

### Slice Preparation and Solutions

Experiments were conducted using brain slices of the VCN obtained from 5- to 12-day-old Sprague-Dawley rats, which were killed by decapitation under sodium pentobarbital (55 mg/kg, i.p.) anesthesia. Each brain was submerged in ice-cold artificial CSF (ACSF) containing (in mM): 124 NaCl, 5 KCl, 1.2 KH_2_PO4, 2.4 CaCl_2_, 1.3 MgSO_4_, 24 NaHCO_3_ and 10 glucose, saturated with 95% O_2_ and 5% CO_2_. The brain region containing the VCN was sectioned into transverse slices (300 μm thick) using a vibratome (VT-1000s, Leica, Germany). Brain slices were pre-incubated in ACSF with 95% O_2_ and 5% CO_2_ for 40–60 min at 35–37°C. The VCN was visually identified in slices with a 40× water immersion objective attached to an upright microscope (ECLIPSE FN1, Nikon, Japan). The neurons with normal morphological appearance and stable electrophysiological activity, i.e., miniature excitatory postsynaptic currents (mEPSCs), evoked EPSCs (eEPSCs) and firings, in the VCN were chosen for experiments.

### Drug Application

The drugs used in this study included bilirubin, β-NAD+, bicuculline, strychnine, 6-Cyano-7-nitroquinoxaline-2,3-dione (CNQX) and D-(-)-2-Amino-5-phosphonopentanoic acid (APV). Chemicals and drugs were all purchased from Sigma unless indicated otherwise. Bilirubin was dissolved in 0.1 M NaOH at 1 mM as a stock solution, stored in disposable aliquots in the dark at −20°C (for <48 h), and diluted to a final concentration of 3 μM before use. Bilirubin was kept away from light both in stock solution and during all experiments. NAD+ was dissolved in distilled water and kept as a stock solution in 10-μL aliquots at −20°C. The NAD solution was used for no more than 5 days after preparation. Tetrodotoxin (TTX) was purchased from Alomone Laboratories (Jerusalem, Israel) and was prepared as concentrated stock solutions in distilled water and stored in 20-μL aliquots at −20°C. The application of drugs was achieved using a multi-valve gravity perfusion system.

### Electrophysiology

#### Whole-Cell Voltage-Clamp Recording of Evoked and Miniature EPSCs

VCN neurons were visually identified using a CCD camera with light filtered to pass visible and infrared. mEPSCs were recorded at a holding potential of −60 mV in the voltage-clamp mode. Pipettes (World Precision Instruments) had a resistance of 3–5 MΩ when filled with an internal solution containing the following (in mM): 127 CH_3_CsO_3_S, 20 NaCl, 20 HEPES, 0.4 EGTA, 5 tetraethylammonium chloride (TEA-Cl), 3 QX314-Cl, 2.5 NaATP and 0.3 GTP. The pH was 7.2 and adjusted with Tris-base. The bath offset potential and electrode capacitance were compensated before sealing onto the cell membrane. The series resistance was compensated by 75%–90%. Series resistance varied from 5 to 15 MΩ among cells in this study and was checked and adjusted every series to keep it below 15 MΩ all along the recordings in one cell. Cells showing the changes in series resistance more than 15% during recordings were omitted from the analysis. Bicuculline (10 μM) strychnine (1 μM) and 1 μM TTX were added to the ACSF to suppress inhibitory currents mediated by GABA, glycine and synaptic currents evoked by action potentials, respectively. To measure evoked EPSCs, a bipolar stimulation electrode was positioned to the auditory nerve stub to stimulate afferent inputs to the recorded cell. Recordings were made 200–400 μm away from the stimulation electrode. The threshold to evoke EPSCs was first measured by varying the stimulation intensity from 0 to 20 V. The stimulus strength was adjusted to be 1.5–2× of threshold in order to elicit reliable minimal EPSCs. Paired-pulses stimulations with inters-pulse time interval of 20 ms were repeated for 15 times with inter-trial interval of 15 s in control, during drug application and wash solutions respectively.

#### Whole-Cell Current-Clamp Recordings of Spike Firings Driven by Presynaptic Release or Current Injections

Pipettes were filled with an internal solution containing the following (in mM): 97.5 K-gluconate, 32.5 KCl, 0.5 EGTA, 40 HEPES, 1 MgCl2, 0.3 GTP and 2 MgATP. The pH was 7.2 and was adjusted with Tris-base. Upon breakthrough of membrane patch, the resting potential of VCN neurons was about −45 to −60 mV. To examine postsynaptic spike firings driven by presynaptic inputs, VCN neurons were held at −70 mV by a hyperpolarization current (−50 to −250 pA) while the auditory nerves were stimulated with a bipolar electrode in a 100 Hz train of five pulses for 15 trials with inter-trial interval of 15 s. The shock intensity was also set at 1.5–2× above the threshold. To examine the intrinsic excitability of VCN neurons, action potentials were elicited from the membrane potential of −70 mV using a series of current injection steps (from −300 to +300 pA with 50-pA increments) in the presence of synaptic blockers of CNQX (40 μM), APV (50 μM), bicuculline (10 μM) and strychnine (1 μM) in ACSF solution.

#### Cell-Attached Recording

Spontaneous action potential currents were recorded from neurons under voltage-clamp mode with a pipette potential of −60 mV. Pipettes with a resistance of 4–6 MΩ were filled with an internal solution containing the following (in mM): 97.5 K-gluconate, 32.5 KCl, 0.5 EGTA, 40 HEPES, 1 MgCl2, 0.3 GTP and 2 MgATP. The pH was 7.2 and was adjusted with Tris-base. All recordings were conducted under a gravity perfusion system at a speed of 1 ml/min of ACSF continuously gassed with 95% O_2_ and 5% CO_2_. All data were acquired by EPC10 (HEKA, 2.9 kHz Bessel filter; LIH 1600 sampled at 10 kHz) with Patch master software or MultiClamp-700B (Axon, 5 kHz low-pass-filtered; 1550, sampled at 50 kHz) with pClamp6 software. All experiments were performed at room temperature (23–27°C).

### Data Analysis

**Figure 1:**
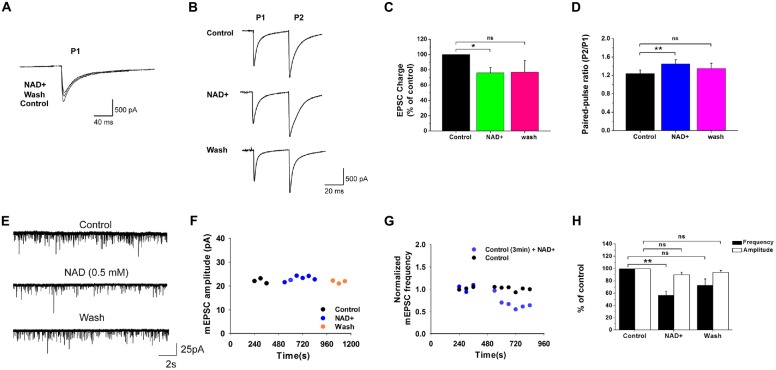
**Effects of nicotinamide adenine dinucleotide (NAD+) on miniature excitatory postsynaptic currents (mEPSCs) and evoked EPSCs. (A)** Evoked EPSCs recorded from a neuron in the presence of strychnine and bicuculline. NAD+ decreased the response charge in this cell. **(B)** EPSCs was evoked in a neuron during paired-pulse stimuli with an interval of 20 ms. NAD+ decreased the first EPSC (P1) but increased the paired-pulse ratio (PPR; P2:P1) in this cell. Stimulation artifacts were manually removed to improve the clarity in **(A,B)**. **(C)** Mean value of normalized charge of the first EPSC in control, during NAD+ and washout solutions. **(D)** Mean value of PPR of EPSC charge in control, during NAD+ and wash solutions. **(E)** Typical mEPSCs recorded from a neuron during control, 0.5 mM NAD+, and washout. **(F)** Time course of average amplitude of mEPSCs along sample experiments of Panel **(E)**. **(G)** Representative time course of the effect of NAD+ on normalized frequency of mEPSCs in a neuron. The control solution was applied for 6 min, followed by the bath application of 0.5 mM NAD+ for 8 min. The frequencies of mEPSCs were measured for last 3 min in control and last 6 min in NAD+ (blue circles). By comparison, in a control experiment the same measurement was made with only vehicle application (black circles). **(H)** Histograms depicting the effect of NAD+ on mEPSCs frequency and amplitude. Error bars represent standard error; *P < 0.05; **P < 0.01; ns: not significant, one-way analysis of variance (ANOVA) with Student-Newman-Keuls *post hoc* test.

All data were stored on a personal computer for further analysis. The numbers of mEPSCs and spontaneous spikes were counted and analyzed with the MiniAnalysis Program (Synaptosoft, NJ, USA), with thresholds 2.5 times the root mean square of the baseline noise, which was typically 6 pA for mEPSCs and 10 pA for spontaneous spike currents. Peaks were detected automatically, but each detected event was then inspected by eye to prevent the inclusion of artifacts. As synaptic charge is less affected by dendritic filtering and series resistance filtering effects, we compared the effect on the first evoked EPSC and on paired-pulse ratio (PPR) by measuring the charge (time integral) of evoked EPSCs to provide a relatively filtering-independent assessment of synaptic conductance. The first spike latency was measured from the onset of a shock to the first peak of the postsynaptic action potential. Action potential threshold was defined as mV at the inflection point on the rising phase. Action potential amplitude was defined as the difference in mV between the peak and threshold. Duration was defined as ms at half maximal amplitude. Rise time was defined as ms of depolarization time from the threshold to the peak. The charge integral of evoked EPSCs and the amplitude of spike latency were analyzed with Clampfit 10.2 (Axon Instruments). The control period was always 6 min in all experiments and the time window used for frequency evaluation was 3 min after the firing activity of the cell reached stability (usually after 3-min recordings). The duration for all drug applications is 8 min and the time window used for frequency evaluation is 6 min after we ensure the drug is working (usually after 2-min recordings). The time for wash was 5 min and the time window used for frequency evaluation is the last 3 min, namely 2 min after switching back perfusion device to the control solution. The average values of mEPSC frequencies and spike frequencies under control conditions were normalized to 1.0 in Figures 1, 3, 5, and were reported as absolute values in the text. The frequency of all synaptic events were provided both as the means ± SEMs and scaled to control values. Statistical analyses were performed using SPSS 17.0 software (SPSS Inc., Chicago, IL, USA). Values in the text and figures are provided as the means ± SEMs. Differences in the frequency and amplitude of the mEPSCs, the charge of evoked EPSCs and the spike latency were evaluated by one-way analysis of variance (ANOVA) with Student-Newman-Keuls *post hoc* test, and the rates of spontaneous spikes were evaluated by Kruskal-Wallis non-parametric test, with *P* < 0.05 used as the level of significance.

## Results

### NAD+ Decreased the Release of Glutamate from Presynaptic Terminals

The VCN can be readily found on the ventral aspect of the inferior peduncle under the microscope. We focused the present study on stellate cells which represent one of the major cell types in the VCN, and can be visually identified from their large soma size and multipolar shape and long dendrites. To determine whether NAD+ affects basal presynaptic glutamate release, we examined evoked EPSCs and mEPSCs from stellate cells before and after the application of 0.5 mM NAD+. In response to two consecutive stimuli to the auditory nerves, these cells showed typical paired-pulse facilitation. Application of NAD+ caused a substantial decrease in the charge of the first eEPSCs to 76% ± 6.8% of control (*n* = 10, *P* < 0.05; Figures 1A,C). When we calculated (PPR = P2/P1), we found NAD+ significantly increase the PPR from 1.2 ± 0.08 to 1.5 ± 0.09 in the presence of NAD+ (*n* = 10, *P* < 0.01; Figure 1D), implying NAD+ can reduce the release probability at the nerve terminal. In line with this interpretation, we found that bath application of NAD+ (0.5 mM) could also significantly decrease mEPSC frequency from 4.6 ± 0.7 Hz to 2.7 ± 0.5 Hz (57% ± 6.1% of the control; *n* = 9, *P* < 0.01) without influencing its amplitude (90% ± 4.2% of the control; *n* = 9, *P* > 0.05). The mEPSC frequency recovered to 3.1 ± 0.4 Hz (73.1 ± 10% of the control) after NAD+ washout (*n* = 9, *P* > 0.05; Figures 1E,F,H). In contrast, five control experiments showed that mEPSCs were stable over the same recording period of time (from 7.7 ± 2.2 Hz to 8 ± 2.6 Hz, 101% ± 8.6% of control; *n* = 5, *P* > 0.05), suggesting that NAD+-induced decrease was not due to run-down of mEPSCs (Figure 1G). These results further indicate that NAD+ suppresses basal presynaptic glutamate release under physiological conditions.

### NAD+ Inhibited the Bilirubin-Induced Potentiation of Presynaptic Glutamate Release

**Figure 2:**
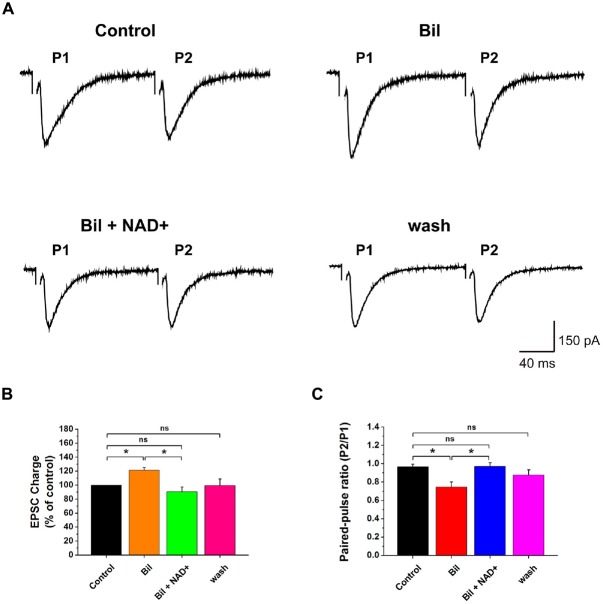
**NAD+ reverses bilirubin induced enhancement in evoked excitatory synaptic transmission. (A)** Evoked EPSCs recorded from a neuron in the presence of strychnine and bicuculline. EPSCs was evoked in a neuron during paired-pulse stimuli with an interval of 20 ms. Bilirubin enhanced the first EPSC (P1) but decreased the PPR (P2:P1) in this cell and the effect was reversed by NAD+. Stimulation artifacts were manually removed to improve the clarity in **(A)**. **(B)** Mean value of normalized charge of the first EPSC in control, bilirubin, bilirubin plus NAD+ and wash respectively and washout solutions. **(C)** Mean value of PPR of EPSC charge in control, bilirubin, bilirubin plus NAD+ and wash solutions respectively. Error bars represent standard error; *P < 0.05; ns: not significant, one-way ANOVA with Student-Newman-Keuls *post hoc* test.

**Figure 3:**
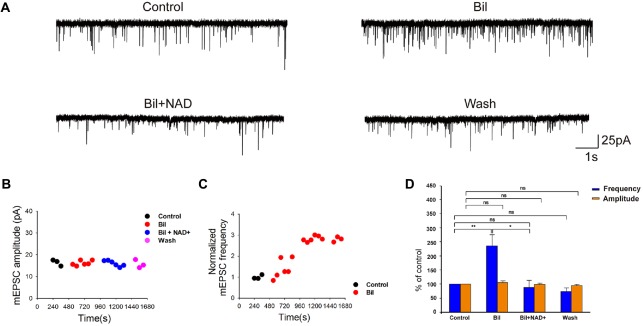
**NAD+ inhibited the bilirubin-induced potentiation of mEPSCs. (A)** Typical mEPSCs were recorded from each neuron for 3, 6, 6 and 3 min time blocks in control, bilirubin, bilirubin plus NAD+ and wash respectively. **(B)** Time course of average amplitude of mEPSCs along sample experiments of Panel **(A)**. **(C)** Representative time course of the effect of bilirubin-induced potentiation of normalized mEPSC frequency in a neuron. Typical time course of mEPSC frequency in a neuron with control period (in black) for 3 min followed by bilirubin application alone (in red) for 21 min is shown. **(D)** Mean values of normalized mEPSC frequencies and amplitudes before, during and after drug administration. Error bars represent the standard error; *P < 0.05; **P < 0.01; ns: not significant, one-way ANOVA with Student-Newman-Keuls *post hoc* test.

Previous studies demonstrated that bilirubin can increase presynaptic glutamate release (Li et al., 2011). To explore whether NAD+ can influence bilirubin-induced potentiation, we recorded the eEPSCs and mEPSCs with sequential application of bilirubin and NAD+. Consistent with the previous results from our laboratory, application of bilirubin caused a substantial increase in the charge of the first eEPSCs to 121.4% ± 9.6% of control (*n* = 7, *P* < 0.05). Subsequent co-application with NAD+ (0.5 mM) could completely reverse the excessive glutamate release induced by bilirubin to 93.6 ± 6.6% of control (*n* = 7, *P* > 0.05; Figures 2A,B). When we calculated PPR, we found bilirubin significantly decrease the PPR from 0.97 ± 0.05 to 0.74 ± 0.03 (*n* = 10, *P* < 0.05). This effect could be partially reversed by NAD+ from 0.74 ± 0.05 to 0.97 ± 0.03 (*n* = 7, *P* < 0.05; Figures 2A,C). We also found that application of bilirubin markedly increased the frequency of mEPSCs from 11.3 ± 7.2 Hz to 17.7 ± 9.9 Hz (235 ± 41% of the control; *n* = 6, *P* < 0.01). Subsequently, co-application with 0.5 mM NAD+ significantly decreased the frequency of mEPSCs from 17.7 ± 9.9 Hz to 6.1 ± 3.4 Hz (88 ± 27% of the control; *n* = 6, *P* > 0.05; Figures 3A,D). In contrast, control experiments showed that the increased frequency in mEPSCs was sustained in the continuous presence of bilirubin, suggesting that NAD+-induced decrease was not due to mEPSC run down and that NAD+ indeed suppressed bilirubin-induced potentiation of glutamate release (Figure 3C). There was no significant change in the mEPSC amplitude throughout the experiment (Figure 3B). These results indicate that NAD+ can inhibit the bilirubin-induced potentiation of presynaptic glutamate release onto stellate cells in VCN.

### NAD+ Decreased the Frequency of Spontaneous Spiking in VCN Neurons

**Figure 4:**
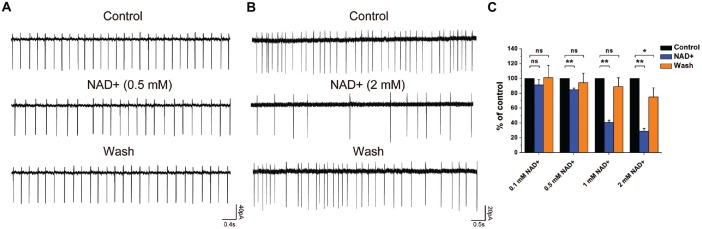
**NAD+ inhibited frequency of spontaneous spikes in ventral cochlear nucleus (VCN) neurons in a dose-dependent manner. (A)** Typical spontaneous spikes were recorded from a neuron in control, NAD+ (0.5 mM), and wash respectively. **(B)** Typical spontaneous spikes were recorded from a neuron in control, NAD+ (2 mM) and wash respectively. **(C)** Group results shows the effect of NAD+ in different concentrations(0.1 mM, 0.5 mM, 1 mM and 2 mM) on the normalized frequency of spontaneous activity. Error bars indicate standard deviation; *P < 0.05; **P < 0.01; ns: not significant, one-way ANOVA with Student-Newman-Keuls *post hoc* test.

To investigate whether NAD+ could influence the neuron excitability, we examined the effect of NAD+ on the frequency of spontaneous spikes in the cell-attached configuration using the voltage-clamp mode. After recording a stable 3-min baseline, NAD+ in different concentrations was applied to the recording brain slice. Only recordings with control firing frequency lower than 6 Hz (80% of cases) were included in the analysis. Figures 4A,B illustrated typical neurons after perfusion of 0.5 mM and 2 mM NAD+. NAD+ application decreased firing frequency significantly (*P* < 0.05). The spontaneous spike frequencies were decreased in a dose-dependent manner from 4.5 ± 1.6 Hz to 4.3 ± 1.5 (91.3 ± 7.3% of the control; *n* = 9, *P* > 0.05) at 0.1 mM, from 5.7 ± 1.0 Hz to 4.7 ± 1.0 Hz (84.6 ± 2.3% of the control; *n* = 9, *P* < 0.01) at 0.5 mM, from 5.3 ± 1.7 Hz to 2.7 ± 1 Hz (40.5 ± 3.2% of the control; *n* = 9, *P* < 0.01) at 1 mM and from 5.2 ± 1.4 Hz to 1.5 ± 0.5 Hz (28.9 ± 3.7% of the control; *n* = 9, *P* < 0.01) at 2 mM, respectively, during 6-min application period (Figure 4C). Thus, these results indicated that neuronal excitability was highly sensitive to NAD+ with a concentration as low as 0.5 mM, being able to induce a significant decrease in the frequency of spontaneous spikes.

### NAD+ Inhibited and Prevented Bilirubin-Induced Hyperexcitation

**Figure 5:**
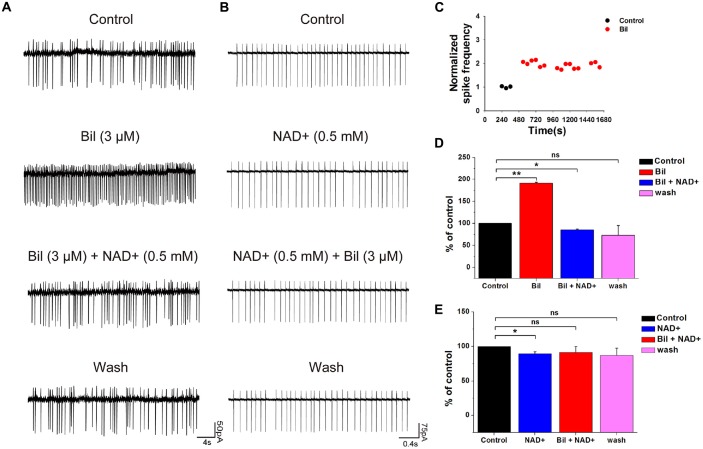
**NAD+ prevented and suppressed bilirubin-induced hyperexcitation. (A)** Typical spontaneous spikes were recorded from a neuron in control solution, bilirubin, bilirubin plus NAD+ and wash respectively. **(B)** Typical spontaneous spikes were recorded from a neuron in control solution, NAD+, bilirubin plus NAD+ and wash respectively. **(C)** Typical time course of normalized spike frequency in a neuron with control solution for 3 min followed by bilirubin application alone for 21 min. **(D)** Mean values of normalized spontaneous spike frequencies of five neurons in control, bilirubin, bilirubin plus NAD+ and wash. **(E)** Mean values of normalized spontaneous spike frequencies of five neurons in control, NAD+, bilirubin plus NAD+ and washout. Error bars represent the standard error; *P < 0.05; **P < 0.01; ns: not significant, Kruskal-Wallis non-parametric tests.

To clarify whether the hyperexcitation induced by bilirubin can be reversed by NAD+, we examined the effect of NAD+ on spontaneous spiking following bilirubin application. The application of bilirubin significantly increased the rate of spontaneous firing from 1.8 ± 1.0 Hz to 4.1 ± 1.9 Hz (191 ± 2.3%, *n* = 5, *P* < 0.01). Subsequent co-application with NAD+ (0.5 mM) could completely reverse the excessive spontaneous firings induced by bilirubin from 4.1 ± 1.9 Hz to 1.8 ± 1.1 Hz (85 ± 2.2% of the control value; *n* = 5, *P* < 0.05). After NAD+ and bilirubin were removed, the frequency rebounded to 1.7 ± 1.1 Hz (73 ± 5.6% of the control; *n* = 5, *P* > 0.05; Figures 5A,D). In contrast, control experiments showed that the frequency of spikes did not decrease in continuous bilirubin application, suggesting that NAD+-induced decrease was not due to cell run down and NAD+ can attenuate bilirubin-induced hyperexcitation (Figure 5C).

Next, to investigate whether NAD+ can prevent bilirubin-induced hyperexcitation, we pretreated recording neurons with 0.5 mM NAD+ before bilirubin application. NAD+ at 0.5 mM significantly decreased the spontaneous spike frequency from 8.2 ± 0.8 Hz to 7.2 ± 0.5 Hz (90 ± 2.8% of the control, *n* = 5, *P* < 0.05). In the presence of NAD+, further addition of bilirubin had no significant influence on frequency of spontaneous spikes (from 7.2 ± 0.5 Hz to 7.2 ± 0.7 Hz, *n* = 5, *P* > 0.05; Figures 5B,E). These results suggest that NAD+ effectively precluded bilirubin-induced hyperexcitation in VCN neurons.

### NAD+ Decreased the Neuronal Intrinsic Excitability

**Figure 6:**
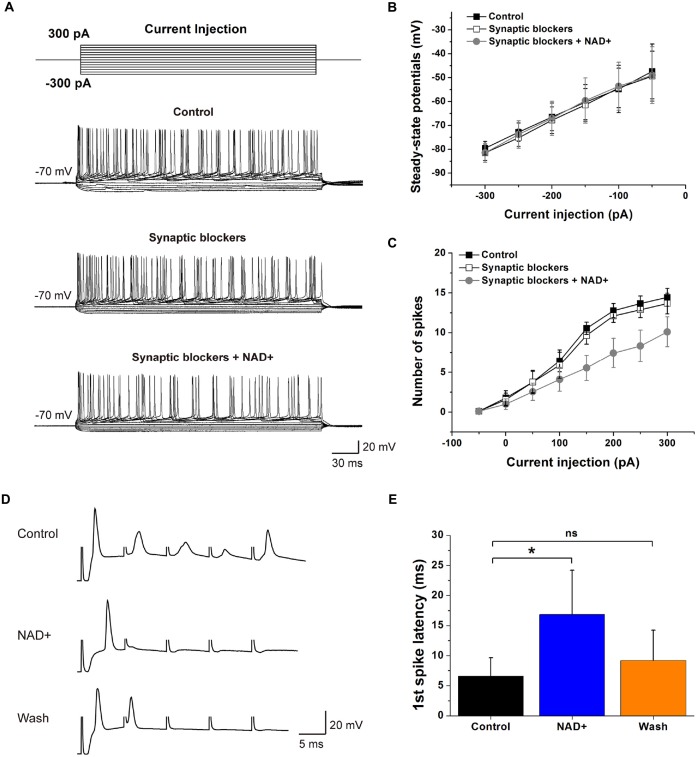
**Effects of NAD+ on neuronal intrinsic excitability and excitatory neurotransmission. (A)** Example traces of APs evoked by depolarizing currents (top panel) from a VCN neuron. Synaptic blockers including 10 μM bicuculline, 1 μM strychnine, 40 μM CNQX and 50 μM APV to block GABA, Glycine, AMPA and NMDA transmitter. **(B)** Voltage-current relations in three conditions. Steady-state potential is measured at the lowest point of the membrane potential in response to hyperpolarizing current steps. **(C)** The number of spikes generated by various depolarization steps from the cells in Panel **(D)**. **(D)** Averaged example traces of neurons in VCN response to auditory nerve shocks. A train of five suprathreshold shocks was delivered to the auditory nerve root at 100 Hz. Trains were repeated 15 times with inter-trial interval of 15 s in control, during NAD+ and wash solutions separately. Stimulation artifacts were manually truncate to for clarity. **(E)** The first spike latency in control (black bar), NAD+ (blue bar) and washout (orange bar) solutions. Error bars represent standard error; *P < 0.05; ns: not significant, one-way ANOVA with Student-Newman-Keuls *post hoc* test.

Our observations showing NAD+ can dampen basal spontaneous spike firings in VCN neurons raised the possibility that the intrinsic excitability of VCN neurons might be dampened, independent its inhibitory effects on presynaptic glutamate release. To test this, we performed current-clamp recordings and examined the input-output relationship of stellate cells by injecting a series of current steps (−300 to +300 pA, 50-pA increments; Figure 6A). Spike numbers at each current step were counted as a measure of the membrane excitability and plotted against the magnitude of current step injections. We found that application of a cocktail of blockers for inhibitory and excitatory receptors (10 μM bicuculline, 1 μM strychnine, 40 μM CNQX and 50 μM APV) had little effects on the input-output curves, suggesting spontaneous transmitter release contributed little to the basal neuronal excitability. In contrast, subsequent addition of NAD+ significantly decreased the number of evoked spikes (*n* = 9), without affecting the input resistance as seen from the steady-state I-V plots measured near the end of current injections (Figures 6B,C), indicating that NAD+ attenuates the intrinsic excitability of VCN neurons. However, the first action potential properties of VCN neurons did not change during the whole period (Table 1).

**Table 1:** **Electrophysiological properties of the first action potential of ventral cochlear nucleus (VCN) neurons**.

	Control	*n*	Synaptic blockers	*n*	Synaptic blockers + NAD+	*n*
AP threshold (mV)	−34.9 ± 1.2	9	−36.7 ± 1.5	9	−38.2 ± 1.6	9	ns
AP amplitude (mV)	67.6 ± 5.6	9	67.4 ± 5.1	9	66.5 ± 5.3	9	ns
AP duration (ms)	1.1 ± 0.1	9	1.3 ± 0.2	9	1.4 ± 0.2	9	ns
AP rise time (ms)	1.1 ± 0.2	9	1.3 ± 0.3	9	1.1 ± 0.2	9	ns

*Data are means ± SEM. ns, not significant*.

The inhibitory effects of NAD+ on the intrinsic excitability of postsynaptic neurons as well as presynaptic release (Figure 1) led us to hypothesize that such dual effects of NAD+ could decrease the fidelity of neurotransmission. To test this, we performed current-clamp recordings of postsynaptic firings driven by presynaptic stimulation with short trains of shocks to the auditory nerve fibers at 100 Hz. As illustrated in Figure 6D, we found that both the latency of the first spike (relative to the stimulation artifact) and total number of spikes were dramatically impacted by NAD+. In line with the effects of NAD+ on EPSCs under voltage-clamp mode (Figure 1), the amplitude of the excitatory postsynaptic potentials (EPSPs) were significantly attenuated to delay the onset of the first spike, as reflected by increases in the latency from 6.5 ± 3 ms to 16.8 ± 7.4 ms after NAD+ application (*n* = 6, *P* < 0.05). This delay was partially recovered to 9.2 ± 5 ms after NAD+ was removed (Figure 6E). All six neurons showed longer AP latency despite that the responses were quite variable among cells. These results suggested that NAD+ potently dampened neuronal excitation by simultaneously attenuating presynaptic glutamate release and postsynaptic intrinsic excitability in VCN.

## Discussion

NAD+ is a ubiquitous molecule with multifaceted biological functions in various biological processes (Ying, 2008). The maintenance of the NAD+ level in the blood or brain is critical for preserving neuronal bioenergetics and promoting cell survival (Wang et al., 2008; Ying, 2008). Different mechanisms by which NAD+ prevents neuronal death have been suggested, but there is a lack of evidence for its effect on neuronal excitation and excitotoxicity associated with many neurodegenerative diseases. In the present study, we explored the effect of NAD+ on neuronal excitation and overexcitation induced by bilirubin in VCN neurons. Our results showed that NAD+ decreased presynaptic glutamate release as evidenced by attenuated charge of eEPSCs and increases in PPR and mEPSC frequency. In parallel, postsynaptic neuronal excitability was also reduced as reflected by the decreased number of neuronal firings in response to the same depolarizing current injections. These data indicated that NAD+ exerts its effects in both pre- and postsynaptic loci in VCN neurons.

The mechanisms underlying the effects of NAD+ on excitatory neurotransmission and membrane excitability remain unknown. It was reported that NAD+ potentiates sodium-activated potassium channels (KNa; Tamsett et al., 2009), which may potentially hyperpolarize the resting membrane potential and attenuate repetitive firings, dampening neuronal excitation. Our results indicate that NAD+ attenuates the intrinsic excitability of VCN neuron by decreasing the number of evoked spikes, raising the possibility that activation of certain channels such as KNa by NAD+ may attenuate repetitive firing instead of individual spike. However, KNa channels are not expressed in these cells (Rothman and Manis, 2003), and neither the resting membrane potential nor the 1st spike waveform was significantly changed by NAD+ (control: −56.7 ± 3.1 mV; NAD+: −57.9 ± 3.3 mV, *n* = 6, *P* > 0.05), making *K*_Na_ an unlikely candidate for NAD+ to exert action. Stellate cells in VCN have low-voltage-activated potassium (IKL) and hyperpolarization-activated (Ih) conductances (Oertel et al., 2008), which may be potential targets for NAD+ to attenuate the neuronal excitability. Further studies are required to identify specific conductances which NAD+ specifically act on.

NAD+ can inhibit both basal synaptic transmission as well as bilirubin-induced increase in glutamate release, suggesting that NAD+ can attenuate glutamate release via unknown mechanisms. Given the fact that bilirubin can induce an increase in intracellular Ca^2+^ (Watchko and Tiribelli, 2013), we speculate that NAD+ acts on either presynaptic Ca^2+^ channels or intra-terminal Ca^2+^ handling to impact the size of eEPSCs and frequency of mEPSCs, but again additional experiments are needed to delineate the effects of NAD+ on release. Nevertheless, our results implicate NAD+ as a potential drug to mitigate the excitotoxicity induced by bilirubin. The present study thus not only unravels the novel effects of NAD+ on the regulation of neuronal excitation under physiological conditions, but also provides the proof-of-principle for the exploration of NAD+ for the prevention and treatment of bilirubin-induced excitotoxicity.

Excitotoxicity is believed to be mediated by the excessive synaptic release of glutamate and the overstimulation of glutamate receptors. The excitotoxicity caused by excessive glutamate is a critical element in the neuropathology of brain disorders (Sattler and Tymianski, 2001). Glutamate excitotoxicity has been linked to many acute and chronic neurodegenerative disorders, such as ischemeia, Parkinson’s disease and bilirubin encephalopathy (Lau and Tymianski, 2010; Li et al., 2011). Glutamate receptor-based therapeutics for excitotoxicity have slowly emerged. Compounds such as MK-801, which blocks glutamate receptors through binding inside the NMDA receptor-associated ion channel and thus prevents Ca^2+^ flux, showed severe CNS-mediated side effects in patients (Foster and Kemp, 2006). A solution to this problem may lie in the development of agents that target other processes of excitotoxicity with fewer side effects. A recent study showed that NAD+ could effectively reduce apoptotic neuronal death after neurons were challenged with excitotoxic glutamate stimulation (Wang et al., 2014). The administration of NAD+ has been shown to have protective effects on ischemeic brain injury, Parkinson’s disease and traumatic brain injury (Ying et al., 2007; Won et al., 2012). Furthermore, the results of the present study demonstrate that NAD+ can reverse the bilirubin-induced glutamate release and neuronal hyperexcitation, and block the bilirubin-induced neuronal hyperexcitation, reinforcing the idea that NAD+ may have preventive and therapeutic potential for managing bilirubin encephalopathy. Suppressing the intrinsic excitability of postsynaptic neurons by NAD+ may further attenuate Ca^2+^ overload during intense activity, expanding its protective action to relieve excitotoxicity in various neurological disorders.

In conclusion, our study demonstrated that NAD+ can markedly decrease basal neuronal excitation as well as over-excitation induced by bilirubin by attenuating presynaptic glutamate release and postsynaptic intrinsic excitability, potentially contributing to its neuroprotective effects. These results provide new information beyond the previously known effects of NAD+ on neuronal function, lending support to NAD+ as a promising agent for both the treatment and prevention of neurotoxicity associated with hyperbilirubinemia and other neurodegenerative disorders.

## Author Contributions

XL-Y and ML: experiments and data collection. WH-Y and HB-S: data analysis and article writing. LY-W, CY-L, SK-Y, NY-S, XL-Y and ML: experiment design and article writing. All authors have reviewed the manuscript.

## Conflict of Interest Statement

The authors declare that the research was conducted in the absence of any commercial or financial relationships that could be construed as a potential conflict of interest.
